# The dual-sensitive period gut-brain crosstalk, neuroinflammation, and the biological roots of adolescent depression

**DOI:** 10.3389/fimmu.2026.1821502

**Published:** 2026-05-25

**Authors:** Yimin Shi, Qian Ma

**Affiliations:** 1 Shandong Provincial Maternal and Child Health Care Hospital Affiliated to Qingdao University, Jinan, China; 2 Jinan Preschool Education College, Jinan, Shandong, China

**Keywords:** adolescent depression, microbiota gut brain axis, microglia, neuroinflammation, sensitive period

## Abstract

The increasing burden of adolescent depression underscores the need to identify developmental vulnerabilities and biologically informed strategies for prevention and intervention. This review synthesizes evidence supporting adolescence as a “dual-sensitive period” during which concurrent maturation of the brain and gut microbiota may create a window of susceptibility to perturbations of the microbiota–gut–brain axis (MGBA). During this stage, dysregulated gut–brain communication may contribute to low-grade inflammation, impaired barrier function, altered HPA-axis responsivity, and changes in tryptophan-, short-chain fatty acid-, and neurotransmitter-related metabolism. In particular, inflammatory signaling and microglial activation may link gut dysbiosis to maladaptive synaptic remodeling, although direct causal evidence in adolescents remains limited. The review also examines the “microgenderome” framework as a lens for understanding potential sex-related differences in depression, and considers environmental exposures such as diet, antibiotics, and sleep disruption as perturbing influences on MGBA homeostasis. Translationally, psychobiotics, dietary optimization, and related microbiota-informed approaches may hold promise as adjunctive or preventive strategies, but they are not yet established standalone treatments for adolescent depression. Future studies integrating longitudinal design, multi-omics profiling, biomarkers, and adolescent-specific intervention trials will be essential to clarify causality and guide precision prevention.

## 1 Introduction

Adolescence, a unique life stage transitioning from childhood to adulthood, is not only a critical period for cognitive and affective development (1) but also a “window of risk” during which the brain undergoes dramatic structural and functional remodeling, rendering it highly susceptible to psychiatric disorders (2). During this period, the prevalence of adolescent depression is escalating at an alarming rate, constituting a severe global public health crisis. Large-scale national surveys from the United States show that between 2009 and 2019 alone, the prevalence of adolescent depression surged from 8.1% to 15.8% (3). This troubling trend is corroborated worldwide, with the Global Burden of Disease (GBD) study indicating a continuously increasing depression burden among adolescents (4), which has become one of the leading causes of disability in this age group (5). The COVID-19 pandemic further exacerbated this crisis, with a global meta-analysis reporting that the prevalence of clinical-level depressive symptoms in adolescents doubled compared to pre-pandemic rates (6). Although psychosocial factors such as the social media environment (7) and adverse childhood experiences (e.g., school bullying) (8) are significant risk factors, these observations raise a fundamental biological question: why is the developing brain so vulnerable, and through what mechanisms are external stressors translated into lasting pathophysiological changes?

The exploration of this question leads directly to the unique characteristics of adolescent brain development. During this period, the brain undergoes profound structural and functional remodeling. Early life stress (e.g., childhood maltreatment) can significantly alter the brain’s developmental trajectory through neuroendocrine and neurotransmitter systems, leaving long-term structural and functional “scars” in regions closely related to emotion regulation, such as the hippocampus, amygdala, and prefrontal cortex, thereby laying a biological foundation for the subsequent onset of psychiatric disorders (9). Modern neuroimaging techniques provide a window to observe these “scars.” A recent multimodal imaging study found that, compared to healthy peers, adolescent depression patients exhibit abnormally enhanced structural-functional connectivity coupling, particularly within the Default Mode Network (DMN). This finding reflects impaired flexibility in information processing in the developing brain and suggests a measurable disturbance in brain network organization (10).

However, in the face of this challenge rooted in brain development, current clinical interventions appear inadequate. Although selective serotonin reuptake inhibitors (SSRIs) are first-line medications, their use in the specific population of adolescents remains associated with uncertainty. An authoritative Cochrane network meta-analysis systematically evaluated the effects of various new-generation antidepressants in children and adolescents, and the findings indicated that, compared to placebo, the efficacy of most drugs is “small and unimportant” (11). Furthermore, a review published in The Lancet Psychiatry critically examined a series of unresolved issues with SSRIs in young people, including activation syndrome, potential suicide risk, and unclear mechanisms of action, underscoring their limited efficacy and non-negligible risks in the developing brain (12). This therapeutic dilemma is not confined to adolescents (13); even for rapidly acting novel drugs like ketamine, safety concerns limit their widespread application (14). Collectively, this clinical evidence suggests that the development of novel intervention strategies with clearer mechanisms and improved safety profiles for the developing brain remains an important unmet need.

Against this backdrop, the microbiota-gut-brain axis (GBA)—an emerging field connecting the environment, genetics, and host physiology—provides a framework for investigating adolescent depression. Depression is increasingly viewed as a systemic disorder sharing biological underpinnings with multiple somatic diseases. The gut microbiome, inflammation, mitochondrial function, and the HPA axis are considered key shared pathways mediating this psycho-somatic comorbidity (15). The complex interplay among diet, microbiota, and depression is thought to occur through these pathways (16). A systematic review focused on adolescent depression further suggested that gut microbiota dysbiosis may be prevalent in depressed adolescents and associated with core symptoms such as anhedonia, while microbiota-targeted interventions have shown preliminary therapeutic potential (17). In addition, human studies in non-adolescent populations have reported reduced gut microbiota diversity in patients with depression (18), and associations between oral microbial pathogens and depressive or anxiety symptoms in adult men have also been described (19). Together, these findings support the possibility that microbial dysbiosis may extend beyond a single anatomical niche and may be relevant to depressive symptomatology across different populations.

The core mechanism of GBA dysfunction lies in the bidirectional “crosstalk” it mediates, in which inflammatory pathways appear to play an important role. Preclinical studies provide preliminary evidence for this proposed pathway: transferring fecal microbiota from depressed patients into germ-free rats induced depression-like behaviors, accompanied by increased inflammatory factors and neuronal alterations in the brain (20). More critically, a study in an adolescent rat model of depression found that modulating the gut microbiota with medication increased the levels of the anti-inflammatory metabolite butyrate in the brain. This, in turn, regulated the function of microglia—the key immune cells of the central nervous system—and was associated with antidepressant-like effects (21). These findings are consistent with the hypothesis that the gut microbiota may act as an upstream regulator of neuroinflammation. Based on this mechanism, GBA-targeted intervention strategies are beginning to move toward clinical exploration, with preliminary trials of probiotics reporting favorable trends in depressive symptoms and inflammatory markers (22). Concurrently, natural products (NPs), with their multi-target and low-toxicity profiles, are being explored as candidate approaches for modulating the GBA and attenuating inflammation (23).

In summary, the epidemic of adolescent depression has become a severe global crisis, and the limitations of current mainstream therapies highlight the urgency of exploring new mechanisms and strategies. Adolescence is not only a period when the brain undergoes critical remodeling and is highly sensitive to early adversity, but it may also represent a sensitive period for the evolution and establishment of a stable gut microbiota community (24–26). Although a growing body of evidence points to the GBA as a potentially important hub linking environmental stress to brain pathology, the dysregulated “crosstalk” between the gut and brain during this specific developmental window—particularly the mechanisms mediated through neuroinflammatory pathways and its potential relevance for early diagnosis and intervention in adolescent depression—remains to be fully elucidated. This is a narrative review rather than a systematic review. Relevant literature was identified through searches of PubMed and Web of Science databases using combinations of key terms including “adolescent depression, ” “gut microbiota, ” “microbiota-gut-brain axis, ” “neuroinflammation, ” “microglia, ” “synaptic pruning, ” “psychobiotics, ” and “dietary intervention, ” with searches conducted up to November 2025. Priority was given to peer-reviewed original research articles, systematic reviews, meta-analyses, and authoritative reviews published within the last ten years, although seminal earlier works were also included. Studies were selected based on their relevance to the intersection of gut microbial ecology, neuroimmunology, and adolescent mental health. As this is a narrative synthesis, formal inclusion/exclusion criteria and risk-of-bias assessments were not applied. Therefore, this review aims to systematically integrate existing evidence, explore the interplay between adolescent brain development and gut microbiota maturation, and propose the following core hypothesis: during the critical developmental window of adolescence, dysregulated crosstalk between the gut microbiota and the brain, mediated through neuroinflammatory pathways, may contribute to remodeling of the developing brain, thereby forming an important biological basis for the high incidence of adolescent depression and representing a potential target for future precision interventions and personalized therapies.

## 2 The “dual-sensitive” developmental window: synchronous maturation of the adolescent brain and gut

A life-course perspective suggests that the development of host physiological systems is not a uniform or linear process, but rather involves multiple “critical windows” or “sensitive periods” during which biological systems are particularly responsive to environmental perturbations (27). During such windows, appropriately timed biological events are required to support normal maturation, whereas exposure to adverse factors such as stress, infection, or nutritional imbalance may produce long-lasting, and in some cases persistent, functional consequences. This idea is often conceptualized as a “double-hit” model (28). Adolescence, as a transitional stage from childhood to adulthood, represents one such sensitive period for mental health trajectories. In this review, we propose the concept of a “dual-sensitive period, ” which we define as the developmental stage during which two critical biological systems—the brain (particularly prefrontal-limbic circuitry involved in emotion regulation) and the gut microbial ecosystem—undergo concurrent, rapid, and interdependent maturation, rendering both systems simultaneously susceptible to environmental perturbations and thereby creating a compounded window of vulnerability for psychiatric disorders, including depression. On one hand, the brain—especially circuits involved in emotion regulation and higher-order cognition—is undergoing extensive remodeling that is strongly influenced by immune and developmental processes; on the other hand, the gut microbial ecosystem is also in a dynamic phase of maturation, shaped by endocrine and environmental changes. These two developmental processes appear to overlap temporally and may interact bidirectionally, forming a potential axis of vulnerability in adolescent depression (29). It should be noted that this conceptual framework is intended as an integrative heuristic to guide future research rather than a fully validated mechanistic model; at present, direct empirical evidence linking the simultaneous maturation of both systems to depression risk in human adolescents remains limited.

### 2.1 The brain as a sensitive period: a highly plastic and vulnerable window of remodeling

The adolescent brain is not simply a smaller version of the adult brain, but rather a dynamic developmental system characterized by the concurrent emergence of advanced cognitive capacities and heightened emotional reactivity (30). One influential framework for understanding this pattern is the neural imbalance model (31). According to this model, subcortical limbic regions involved in emotion, motivation, and reward processing—such as the amygdala and ventral striatum—mature earlier than the prefrontal cortex (PFC), which supports cognitive control, decision-making, and emotion regulation. This developmental asynchrony between a relatively earlier “accelerator” system and a still-maturing “brake” system may contribute to increased sensitivity to emotional and social stimuli during adolescence (31) and may also create a window of vulnerability for mood disorders, including depression (29, 30).

This macroscopic functional imbalance is underpinned by extensive microscopic structural remodeling. One of the most prominent features of adolescent brain development, particularly within the PFC, is large-scale synaptic pruning (33). During this process, gray matter volume follows an inverted U-shaped trajectory, peaking in late childhood and declining throughout adolescence, as redundant or less efficient synaptic connections are selectively eliminated to improve the efficiency and specificity of neural networks—a pattern initially demonstrated through postmortem synaptic density measurements (33) and subsequently confirmed by longitudinal structural neuroimaging studies. Synaptic pruning is essential for cognitive maturation, but it may also represent a vulnerable biological juncture. Genetic or environmental risk factors, including immune activation, may disturb this process and lead to either insufficient or excessive pruning, thereby altering cortical circuit function. Such dysregulated pruning has been implicated as a potential mechanism in several psychiatric disorders; for example, the synaptic hypothesis of schizophrenia proposes that excessive complement-mediated pruning contributes to cortical gray matter loss (32), and analogous, though less well-characterized, processes may be relevant to depression (35).

Microglia, the resident immune cells of the central nervous system, play a key role in the execution of synaptic pruning (36). During adolescence, microglia are themselves in a developmentally active state and contribute to the refinement of neural circuits in a process that has been referred to as “immunoadolescence” (34). At the same time, white matter pathways supporting long-range connectivity continue to mature. Recent systematic reviews indicate that pubertal sex hormone changes are associated with widespread remodeling of white matter microstructure, including myelination and axonal organization, particularly in structures such as the corpus callosum (35, 36). These structural changes are reflected in functional development. For example, emotion regulation does not improve in a strictly linear manner across adolescence; instead, some studies suggest a transient decline in affective control during mid-adolescence, which may help explain the heightened emotional vulnerability observed in this age group (37, 38).

It should be acknowledged, however, that the precise timing and extent of these neurodevelopmental processes show considerable interindividual variability, influenced by sex, pubertal tempo, genetic background, and socioeconomic factors. Moreover, most mechanistic understanding of synaptic pruning derives from rodent models or postmortem human studies, and the degree to which these findings directly reflect *in vivo* adolescent brain remodeling in real time remains an area of active investigation.

The concurrent remodeling of cortical structure, immune regulation, and white matter connectivity during adolescence suggests that this period represents a neurobiologically sensitive window. In the following section, we examine whether a parallel sensitive window exists for the gut microbial ecosystem, and whether these two developmental trajectories may interact.

### 2.2 The gut as a sensitive period: a dynamic and unstable microbial ecosystem

In parallel with brain remodeling, the gut microbiome also undergoes substantial developmental changes during early life and adolescence. Large longitudinal studies have characterized microbial succession during infancy and early childhood in detail. For example, the TEDDY study identified distinct phases of early microbial development, including a developmental phase dominated by Bifidobacterium, followed by a transitional phase with increasing diversity and then a stabilizing phase as the microbiota approaches a more mature configuration (39). Early colonization is important for immune system education and long-term health, and disturbances during this period—including delivery mode, feeding pattern, and antibiotic exposure—can have lasting effects (40).

Importantly, microbial development does not end in childhood. Accumulating evidence suggests that adolescence may represent a second important window during which the gut microbiota continues to change (41). A study comparing prepubertal and pubertal children reported differences in bacterial composition and abundance, together with associations between certain genera and sex hormone levels such as testosterone, suggesting that pubertal endocrine changes may directly shape microbial community structure (42). In addition, recent research and reviews indicate that microbial richness and community composition continue to evolve throughout childhood and adolescence, with dietary patterns playing a particularly influential role during this developmental stage (43). Taken together, these findings suggest that, in response to pubertal endocrine changes and shifting environmental exposures, the gut microbial ecosystem remains highly plastic during adolescence and may therefore be particularly sensitive to influences such as diet, stress, and medication exposure (44).

Nevertheless, several important limitations should be noted. Most studies of the adolescent gut microbiome are cross-sectional, making it difficult to distinguish true developmental trajectories from cohort effects. Large-scale longitudinal studies tracking gut microbial composition across the pubertal transition in conjunction with clinical outcomes remain scarce. Furthermore, much of the existing data comes from Western populations, and the generalizability of these patterns across diverse dietary and cultural contexts is uncertain.

Having established that both the brain and the gut microbial ecosystem undergo dynamic remodeling during adolescence, we now turn to the question of whether perturbations in one system may influence the developmental trajectory of the other—a possibility that forms the core of the dual-sensitive period hypothesis.

### 2.3 The dual-sensitive period model: linking gut dysbiosis to aberrant brain development

Given the synchronous sensitive periods of the brain and gut during adolescence, a central hypothesis emerges: perturbations in the gut microbial ecosystem may alter signaling through the microbiota-gut-brain axis (45) and thereby interfere with ongoing neurodevelopmental processes, including synaptic pruning in the PFC. Such interference may contribute to abnormal maturation of emotion-regulation circuits and increase the risk of depression. In this context, the “double-hit” model proposes that an early-life insult—such as adverse microbial colonization—may create a vulnerable biological background, whereas a second insult during adolescence—such as stress or an unhealthy diet leading to dysbiosis—may precipitate pathological outcomes (28). Although this model remains only partially validated in humans, it is increasingly supported by preclinical evidence (28).

Accumulating evidence from animal models demonstrates that the gut microbiota can directly influence brain function and behavior (46). Importantly, the host microbiota has been shown to constantly control the maturation and function of microglia in the central nervous system (47), suggesting a direct mechanistic link between gut microbial composition and the neural remodeling processes that occur during adolescence. Several recent preclinical studies provide further mechanistic support for this hypothesis. One study using a chronic stress paradigm in adult mice showed that stress-induced gut dysbiosis can promote abnormal microglia-mediated synaptic pruning in the PFC by activating peripheral and central complement C3 signaling, ultimately leading to depression-like behaviors; notably, transplantation of fecal microbiota from stressed mice into germ-free mice was sufficient to reproduce these pathological changes (48). Another study, using a model of intrauterine growth restriction in rodents, identified a different pathway: gut dysbiosis reduced the gut-derived metabolite indole-3-propionic acid (IPA), which in turn impaired activation of the aryl hydrocarbon receptor (AHR) in the brain, and this alteration was associated with excessive microglial synaptic pruning and social behavior deficits; both IPA supplementation and fecal microbiota transplantation from healthy donors were able to partially reverse these phenotypes (49). These findings suggest that immune-related signals, such as complement activation, and metabolic signals, such as reduced IPA, may both contribute to disrupted brain remodeling through gut-derived mechanisms. However, it is essential to note that these observations are based entirely on rodent models, often using adult animals or perinatal paradigms, and their direct applicability to adolescent depression in humans remains to be established. Whether these specific pathways operate during the human pubertal window, and whether the effect sizes observed in controlled animal experiments translate to clinically meaningful impacts in the more complex human context, are questions that require dedicated investigation.

**Figure 1:**
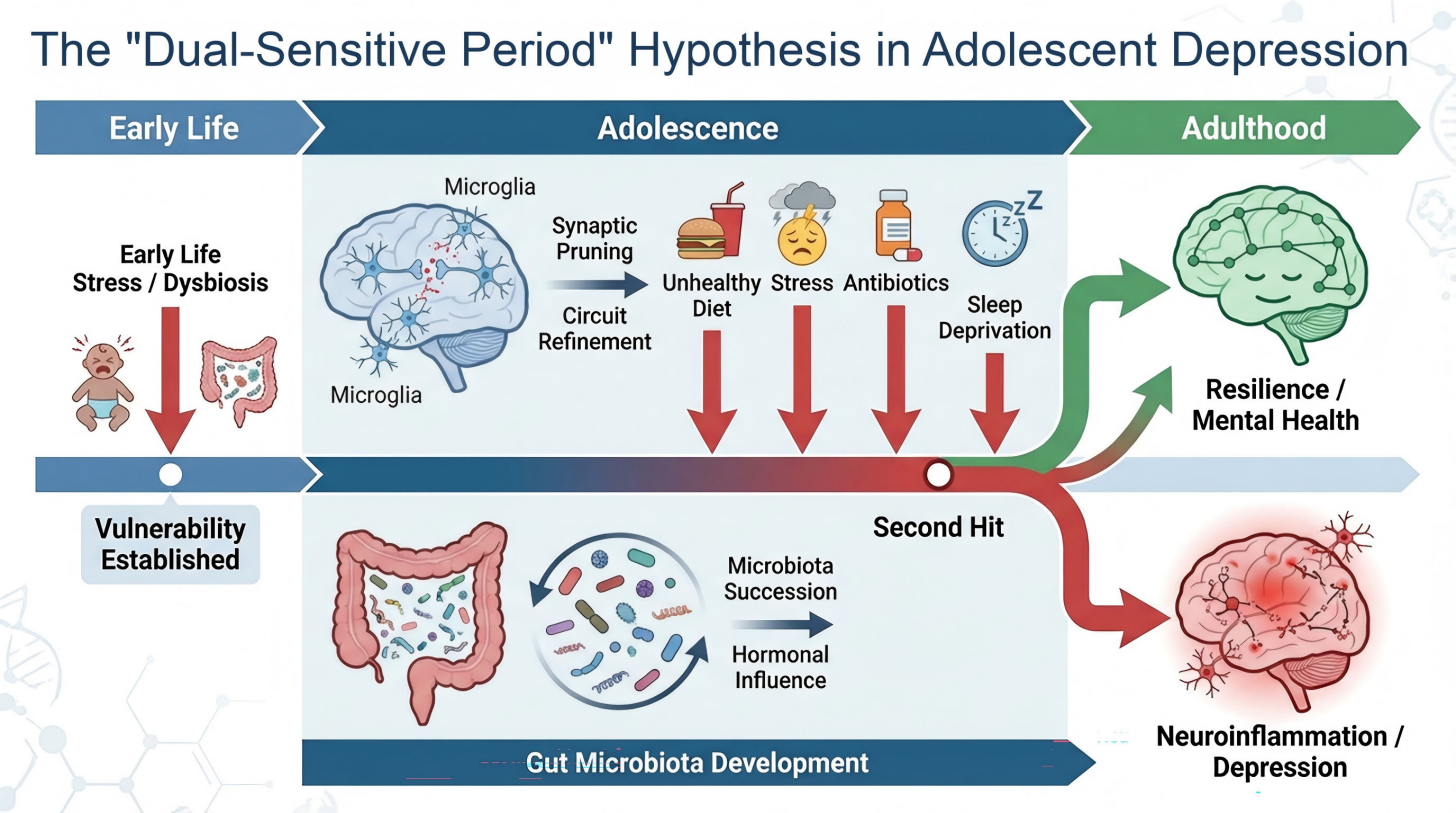
Proposed dual-sensitive period model for adolescent depression. The figure summarizes the hypothesis that adolescence represents a dual-sensitive developmental window characterized by concurrent maturation of the brain and gut microbiota. Environmental first hits occurring early in life may prime vulnerability, whereas second hits during adolescence—including stress, diet, antibiotics, and sleep disruption—may disrupt microbiota–gut–brain axis homeostasis. These disturbances may converge on inflammation, barrier dysfunction, microglial activation, and aberrant synaptic pruning, thereby increasing susceptibility to depression.

In summary, adolescence may represent a biologically distinct dual-sensitive period in which brain remodeling and gut microbial maturation occur in parallel. This overlapping developmental window may amplify vulnerability to environmental perturbations and thereby influence the trajectory of mental health. The following chapters will examine the specific molecular and cellular mechanisms—including neuroinflammatory pathways, neuroendocrine signaling, and sex-specific factors—through which the gut microbiota may interact with the developing brain during this critical period (**Figure 1**).

## 3 Mechanisms of action

Adolescence represents a biologically vulnerable window in which the brain and the gut microbiota undergo partially overlapping maturation. Within this dual-sensitive period, perturbations of gut microbial homeostasis may exert disproportionate effects on brain development and stress regulation, thereby increasing susceptibility to depression. Current evidence suggests that gut-brain communication during this period is mediated by three interrelated pathways: immune and neuroinflammatory signaling, vagal and neurotransmitter-related signaling, and hypothalamic-pituitary-adrenal (HPA) axis regulation (50, 51). Rather than functioning independently, these pathways likely converge on shared downstream processes, including microglial activation, synaptic remodeling, and altered stress responsivity. However, it should be emphasized that much of the mechanistic evidence still derives from animal models or adult studies, and adolescent-specific human data remain limited.

### 3.1 The immune and neuroinflammatory pathway: microglia-mediated synaptopathy

Neuroinflammatory dysregulation is increasingly recognized as a potential contributor to depression, including in adolescent populations, although the strength of direct human evidence remains modest (52). The gut may serve as an upstream trigger for this process. Chronic stress, dietary imbalance, and sleep disruption can impair intestinal barrier integrity and promote a “leaky gut” state (50). Under these conditions, microbe-associated molecular patterns (MAMPs), including lipopolysaccharide (LPS) derived from gram-negative bacteria, may enter the systemic circulation and contribute to low-grade peripheral inflammation (51, 52).

Peripheral inflammatory signals such as LPS and pro-inflammatory cytokines, including TNF-α and IL-1β, may then influence the brain through multiple routes, including altered blood-brain barrier (BBB) permeability and immune-to-brain signaling pathways (53, 54). These signals can promote microglial activation, which is particularly relevant during adolescence because microglia are actively involved in circuit refinement and synaptic pruning during this developmental stage (55). Under physiological conditions, this pruning process supports the maturation of neural networks. However, in the context of chronic inflammation or stress, microglia may become overactivated and contribute to excessive synaptic elimination, potentially disrupting fronto-limbic connectivity and emotional regulation (56).

**Figure 2:**
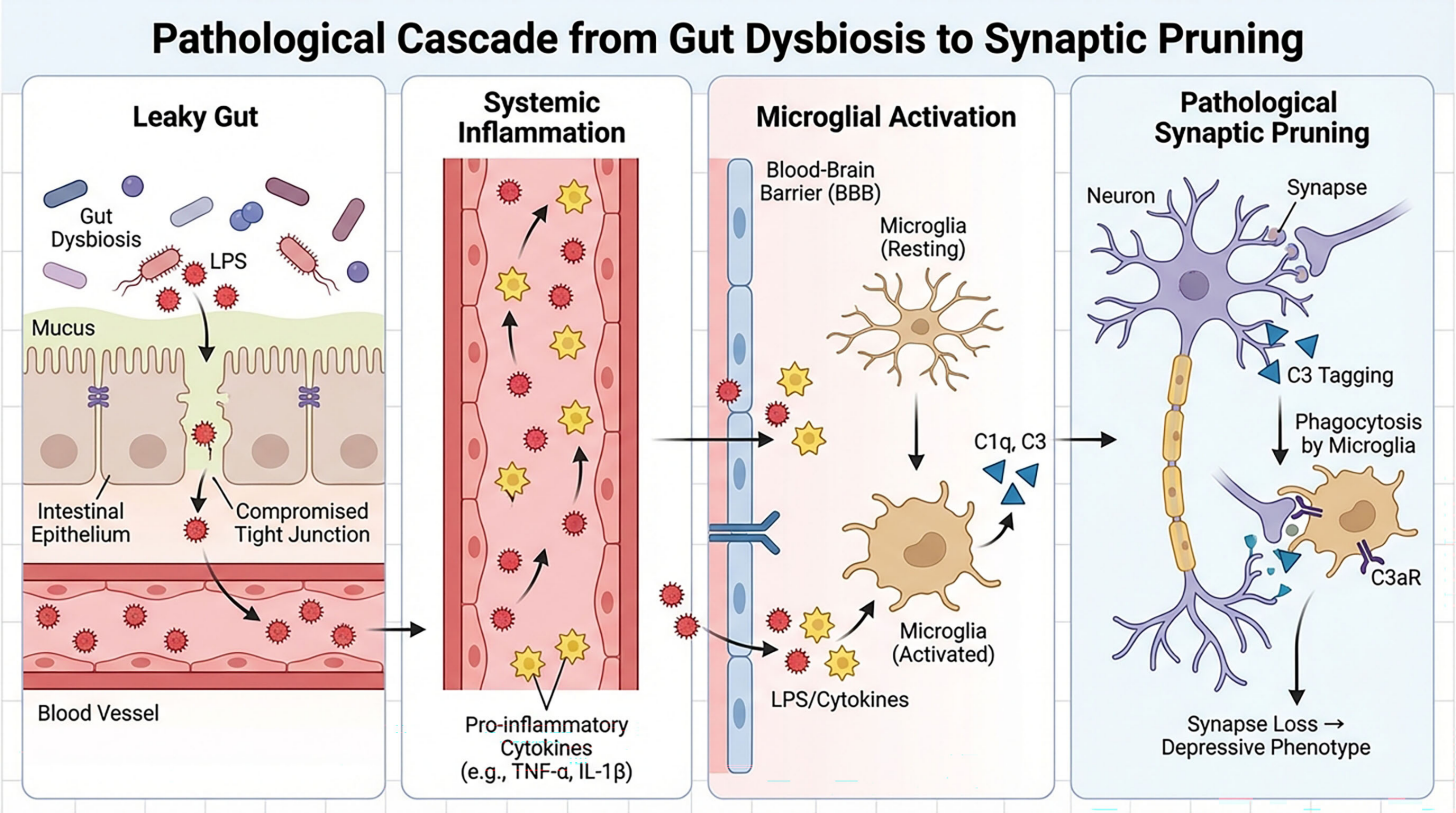
Mechanistic cascade linking gut dysbiosis to depressive pathology through neuroinflammatory signaling. Gut dysbiosis may increase intestinal permeability and facilitate translocation of microbial products, such as LPS, into the circulation. Peripheral inflammatory signals can compromise blood–brain barrier integrity and activate microglia. Activated microglia and related complement signaling pathways may promote excessive synaptic tagging and phagocytic pruning, leading to disruption of developing neural circuits involved in emotion regulation and the emergence of depressive symptoms.

Several experimental studies have begun to define molecular links between inflammation and synaptic remodeling. For example, inflammatory stimuli such as LPS may induce high mobility group box 1 (HMGB1)-related signaling, which in turn can promote complement pathway activation, including C1q and C3 expression (57). In parallel, IL-1–responsive astrocytic signaling has been implicated in complement C3-mediated synaptic tagging and microglial pruning (58). Microglia can recognize complement-tagged synapses via receptors such as CR3 and C3aR and then remove them through phagocytosis (45, 58). In rodent models, manipulation of the gut microbiota has also been reported to alter microglial inflammatory activity and depressive-like behavior. For instance, rifaximin treatment in adolescent rats increased brain butyrate levels, reduced microglial pro-inflammatory signaling, preserved hippocampal neurogenesis, and alleviated depressive-like behaviors (21). These findings support a mechanistic link between gut dysbiosis, inflammation, and synaptic dysfunction in adolescent animal models, although direct confirmation in human adolescents is still lacking. Accordingly, interventions targeting this cascade, including modulation of HMGB1/C1q/C3-related signaling or complement receptor pathways, may represent promising therapeutic strategies, but their translational relevance requires further validation (57, 59) (**Figure 2**).

### 3.2 The vagus nerve and neurotransmitter pathway: gut-derived metabolite-mediated neuromodulation

In addition to humoral immune signaling, the vagus nerve provides a rapid bidirectional communication route between the gut and the brain (47, 48). Approximately 80% of vagal fibers are afferent, allowing the central nervous system to receive peripheral information related to microbial metabolites and gut-derived signals via the nucleus of the solitary tract and connected brainstem circuits. Experimental vagotomy studies have shown that some microbiota-dependent behavioral effects require an intact vagus nerve. For example, fecal microbiota transplantation from stressed donors induced depressive-like behavior in recipient mice, whereas this effect was attenuated when the vagus nerve was severed (60). Similarly, the behavioral effects of Lactobacillus reuteri in preclinical models have been shown to depend on vagal integrity (61). In a preliminary translational study, a randomized controlled trial reported that multi-species probiotic supplementation enhanced vagal nerve function in patients with depression, providing early human evidence that microbial interventions may modulate this pathway (62). These findings suggest that vagal signaling may be one route through which gut microbial changes influence affective behavior, although direct evidence in adolescents is still sparse.

Gut microbiota may also influence brain function through the production and regulation of neuroactive metabolites. Tryptophan metabolism is particularly relevant because tryptophan is an essential precursor for serotonin synthesis. Under inflammatory conditions, indoleamine 2, 3-dioxygenase (IDO) activity may increase, diverting tryptophan away from serotonin production toward the kynurenine pathway and generating metabolites with potentially neurotoxic or pro-oxidative effects (16). In adolescent depression, emerging preclinical work suggests that these metabolic shifts may be important. One study reported that transplantation of Roseburia hominis attenuated adolescent stress-induced gut dysbiosis and depressive-like behaviors in mice, with effects linked to reduced IDO1 expression, suppression of kynurenine pathway activity, and increased brain serotonin levels (63). Another study identified Coprococcus eutactus, isolated from healthy adolescents, as a potential antidepressant-associated bacterium that alleviated chronic restraint stress-induced depressive-like changes in adolescent mouse models, possibly through modulation of neurotransmitter pathways (64). In addition, gut bacteria-driven production of homovanillic acid has been reported to alleviate depressive-like behavior by modulating synaptic integrity in preclinical systems (65). Taken together, these findings suggest that gut microbial signaling may shape brain chemistry and synaptic function through both neural and metabolic routes. Nevertheless, most available evidence remains preclinical, and whether these mechanisms operate in the same way during human adolescence remains to be established.

### 3.3 HPA axis hyperreactivity: dysregulation of the stress response rhythm

The HPA axis is a central endocrine system that coordinates responses to stress, and adolescence appears to be a particularly sensitive period for stress-axis recalibration (66). The gut microbiota is increasingly recognized as a modulator of HPA axis function. Studies in germ-free animals have shown that the absence of a normal microbiota is associated with exaggerated stress hormone responses, indicating that early microbial colonization contributes to HPA axis development and calibration (48). More recent work suggests that microbiota-related effects on stress responsivity may involve circadian regulation rather than simple hormone elevation alone. In experimental models, microbiota depletion has been associated with disruption of the normal daily rhythm of corticosterone release, alongside altered clock-gene expression in limbic and circadian brain regions (66). These findings imply that gut dysbiosis may impair stress adaptation by disturbing the temporal organization of endocrine signaling, which is conceptually relevant to sleep disturbance and diurnal mood variation in adolescent depression.

Microbial metabolites such as short-chain fatty acids (SCFAs), including butyrate, may contribute to this regulation through multiple mechanisms, including enhancement of gut barrier integrity, modulation of immune signaling, and indirect effects on neural circuits via the vagus nerve (21). In addition, dietary exposures during developmental periods may shape both microbiota composition and stress responsivity (67), although direct evidence linking these effects specifically to HPA axis programming in adolescents remains limited (68). Overall, available data indicate that gut microbial homeostasis may contribute to the establishment of resilient stress regulation during adolescence, but the extent to which these findings translate to human adolescent depression is still uncertain.

## 4 Key modulating variables: sex hormones and the microgenderome

### 4.1 The “microgenderome”: a conceptual framework for understanding sex differences

After puberty, the epidemiological pattern of depression changes markedly, with females showing approximately twice the prevalence of males across many populations (69). This sex difference has been observed across cultural settings, although its biological, developmental, and social determinants likely interact in complex ways. In depressed children and adolescents, alterations in stress-related neuroendocrine function, particularly involving the hypothalamic-pituitary-adrenal (HPA) axis, have been reported, suggesting that neuroendocrine mechanisms may contribute to vulnerability during this developmental period (70). Given the central role of the gut-brain axis in stress responsivity and emotion regulation, sex-dependent variation within this system may represent one contributor to post-pubertal differences in depression risk (71).

To describe this bidirectional interaction, the term “microgenderome” has been proposed as a conceptual framework encompassing sex hormones, the gut microbiota, and host immune function (72). Within this framework, sex hormones may shape microbial composition and activity, whereas the gut microbiota may participate in hormone metabolism and signaling, thereby contributing to sex-specific physiological and pathological outcomes (73). One important mechanistic component is the “estrobolome, ” a subset of gut microbes capable of producing β-glucuronidase, which can deconjugate estrogen metabolites and influence enterohepatic estrogen recycling (74, 75). Human cross-sectional evidence has linked systemic estrogen profiles with fecal microbiota diversity and β-glucuronidase gene richness, providing early support for the estrobolome concept (76). Experimental work also suggests that changes in estrogen status can alter microbial structure and function, including β-glucuronidase-related activity (77). However, most direct evidence for this pathway comes from adult or animal studies, and its relevance to adolescent depression remains inferential rather than fully established (78).

### 4.2 Core mechanisms: sex dimorphism in stress response and barrier function

The microgenderome hypothesis is particularly relevant to stress-related psychiatric disorders because stress responses are often sex-dependent. Available evidence suggests that sex-dependent stress biology may partly contribute to the higher post-pubertal burden of depression observed in females (79). Puberty is a period of substantial hormonal change, and these changes may interact with stress responsivity in a sex-specific manner (80). Human evidence indicates that acute psychosocial stress can rapidly alter small-intestinal mucosal microbiota composition, with evidence of sex-dependent microbial shifts (81). Consistent with this broader pattern, rodent studies suggest that chronic stress may produce sex-dependent behavioral and neurobiological outcomes, including greater vulnerability to anxiety- or depressive-like phenotypes in females under some stress paradigms (82). Stress exposure during puberty may also exert long-lasting, sex-specific effects on depressive-like behavior and monoamine neurotransmitter levels that persist into adulthood, highlighting puberty as a sensitive developmental window for stress-related programming (83).

A plausible mechanistic node linking sex hormones, stress vulnerability, and gut-brain communication is barrier function. Emerging evidence supports a conceptual framework in which sex hormones may coordinately influence both intestinal barrier integrity and blood-brain barrier (BBB) stability, thereby modulating inflammatory access to the central nervous system (84–89). At the intestinal level, estrogen-related pathways appear to exert protective effects on epithelial integrity under inflammatory or stress-related conditions. Experimental models indicate that estrogen replacement can attenuate stress-induced intestinal permeability and colonic inflammation, while 17β-estradiol treatment can reduce colonic permeability and inflammatory cytokine expression in colitis models (84, 85). Estrogen receptor β has also been implicated in colonic epithelial homeostasis and inflammatory responses, supporting a potential role for estrogen signaling in maintaining mucosal integrity (86). Clinical evidence further suggests that chronic stress exposure may be associated with sex-specific alterations in jejunal tight junction proteins and microbiota composition, with greater barrier disruption observed in females in some contexts (87).

Similar sex-hormone-related modulation may occur at the BBB. Estrogen has been associated with reduced inflammation-induced BBB disruption, lower endothelial adhesion molecule expression, and decreased immune-cell trafficking across the barrier (88). Conversely, androgen depletion has been linked to BBB dysfunction, increased neuroinflammatory markers, and disruption of tight junction-related proteins in male animal models (89). Together, these findings suggest that sex hormones may shape stress vulnerability through coordinated effects on intestinal and central barrier systems. Nevertheless, direct evidence connecting these mechanisms to adolescent depression in humans remains limited, and developmental-stage-specific studies are needed.

### 4.3 Mechanistic integration and clinical implications

The microgenderome framework provides a useful, but still provisional, lens for understanding sex differences in adolescent depression. During puberty, changing levels of sex hormones may reshape microbial composition and function in a sex-dependent manner, while stress exposure may amplify downstream effects on immune signaling, barrier integrity, and neuroactive metabolite production (90–92). Preclinical studies provide mechanistic plausibility for this model: estrogen-deficiency or reproductive-state-related models of depressive-like behavior have been associated with gut dysbiosis, altered tryptophan metabolism, hippocampal NLRP3-mediated neuroinflammation, and systemic inflammatory changes (90–92). In some models, microbial or microbiota-targeted interventions appear to attenuate depressive-like phenotypes, further supporting a potential role for the gut microbiota in hormone-related mood vulnerability (90, 92). However, most of these findings derive from adult or animal models, and they should be interpreted as indirect evidence rather than direct proof of equivalent processes in adolescents.

Importantly, microgenderome-related processes should not be viewed as the sole explanation for sex differences in depression. Genetic sex chromosome effects may contribute substantially, with evidence from sex chromosome aneuploidies indicating that X-linked gene dosage can influence psychiatric vulnerability independently of gonadal hormone exposure (93). Puberty-related epigenetic remodeling of neural circuits may also play a critical role, as longitudinal methylation evidence suggests that sex-specific DNA methylation changes emerging during puberty may predict later depressive symptoms (94). Therefore, the microgenderome should be regarded as one component of a broader developmental framework rather than a comprehensive explanation. Future studies in adolescents, especially longitudinal and sex-stratified designs, are needed to test whether this conceptual model can be translated into clinically meaningful biomarkers or interventions (95, 96).

## 5 Environmental triggers: perturbers of the gut-brain axis

Adolescence is a sensitive developmental period during which environmental exposures may influence both gut microbial ecology and brain function. Because the gut microbiota continues to interact with immune, endocrine, and neural maturation during this stage, modifiable lifestyle-related factors may shape vulnerability to depressive symptoms and related neurobehavioral outcomes. Among these factors, dietary patterns, antibiotic exposure, and sleep or circadian disruption are common in contemporary adolescent life and have been associated, through partly overlapping pathways, with gut dysbiosis, low-grade inflammation, metabolic disturbance, and altered stress responsivity (97). This chapter therefore focuses on these three major environmental triggers as potentially important perturbing influences on the adolescent gut-brain axis.

### 5.1 Dietary patterns: a catalyst for pro-inflammatory states

Diet is one of the most direct environmental determinants of gut microbiota composition and metabolic output. In adolescents, habitual consumption of high-fat, high-sugar Western-style dietary patterns has been associated with a more pro-inflammatory physiological milieu and a higher risk of depressive symptoms, whereas Mediterranean-style dietary patterns rich in fiber, polyphenols, and unsaturated fatty acids appear to exert protective effects through anti-inflammatory and microbiota-supportive actions (97).

Experimental studies provide mechanistic support for these associations. Adolescent exposure to obesogenic or cafeteria-style diets can induce persistent shifts in gut microbial composition and may be accompanied by long-term changes in brain regions involved in emotional processing, including altered expression of neuroimmune- and neurotransmission-related genes in the amygdala (98). These findings suggest that dietary exposures during adolescence may leave lasting microbiota- and neuroimmune-related signatures that extend beyond the period of active exposure. Evidence from developmental models further indicates that vulnerability may begin even earlier: maternal high-fat diet can alter maternal microbiota and metabolism, including pathways related to kynurenine metabolism, with downstream effects on embryonic brain metabolites and adolescent behavioral phenotypes in offspring (99).

Taken together, current evidence supports unhealthy dietary patterns as an important environmental risk factor for emotional dysregulation and depression-related outcomes across development. Proposed mechanisms include microbiota disruption, increased intestinal permeability, immune activation, neuroinflammation, and altered HPA-axis signaling (100). In addition to these inflammatory and neuroendocrine pathways, metabolic and vascular mechanisms may also contribute to broader neurobehavioral outcomes. Dietary fat intake has been linked to variation in VEGF- and GLUT1-related pathways relevant to cerebrovascular homeostasis, glucose transport, and cognitive function, although these findings are more directly related to neurocognitive and vascular-metabolic pathways than to adolescent depression specifically (101).

### 5.2 Antibiotic exposure: microbiota perturbation during a critical window

Antibiotic exposure is another major environmental factor capable of disrupting gut microbial development during sensitive periods. Human observational evidence suggests that prolonged antibiotic use in early life is associated with later anxiety, depression, and poorer cognitive performance, and that these associations may interact with individual genetic susceptibility (102). Such findings are biologically plausible given the central role of the microbiota in neuroimmune maturation and brain development.

Animal studies further support the existence of developmental windows of heightened vulnerability to antibiotic-induced dysbiosis. Microbiota disruption during early postnatal life or around weaning has been shown to produce persistent alterations in gut microbial communities, peripheral immune profiles, and neurodevelopment-related processes, including myelination and microglial morphology (103). Similarly, comparative depletion studies indicate that microbiota disturbance during infancy and adolescence may produce more pronounced long-term cognitive and emotional abnormalities than similar interventions initiated in adulthood (104). These data support the view that adolescence, together with earlier developmental stages, represents a period of increased sensitivity to microbial perturbation (105).

At the same time, the relationship between antibiotics, microbiota, and depression is unlikely to be unidirectional across all contexts. In stress-based adolescent models, targeted modulation of the gut microbiota with a non-absorbable antibiotic such as rifaximin has been reported to reduce microglial inflammatory activation and alleviate depression-like behavior, supporting a contributory role of gut microbes in stress-related neuroinflammatory pathways (21). However, translation to human populations remains complex. Large-scale epidemiological evidence suggests that treated infections and anti-infective exposure in childhood and adolescence may be associated with subsequent risk of treated mental disorders, but confounding by infection severity, indication, familial factors, and shared environmental influences remains difficult to exclude (106). Consistent with this complexity, clinical studies in adolescents with depression have not uniformly identified significant compositional differences in the gut microbiota relative to healthy controls (107). These inconsistencies highlight the need for well-controlled longitudinal studies integrating exposure timing, antibiotic class, host genetics, diet, infection burden, and functional microbial readouts.

### 5.3 Sleep deprivation and circadian disruption: disruptors of biological rhythm

Insufficient sleep and circadian disruption are widespread in modern adolescents and are increasingly recognized as modulators of the gut-brain axis. Sleep and circadian processes are bidirectionally linked to the gut microbiota, and their disruption may promote gut dysbiosis, impaired epithelial barrier integrity, HPA-axis activation, and systemic inflammation (108, 109). In human adolescents, objectively measured sleep characteristics have been associated with gut microbial diversity and composition, supporting the relevance of this relationship in real-world developmental settings (110).

Mechanistic evidence from animal models has strengthened this link. Sleep deprivation can induce anxiety- and depression-like behaviors together with gut microbial disturbances, increased intestinal permeability, and peripheral or central inflammatory responses (111). Fecal microbiota transplantation studies further support a contributory role of the microbiota: transfer of microbiota from sleep-deprived donors to microbiota-depleted or germ-free recipients can reproduce neuroinflammatory and cognitive abnormalities, with inflammatory signaling pathways such as TLR4/NF-κB implicated in this process (111).

Emerging evidence also suggests that sleep loss may influence developmental trajectories beyond mood symptoms. For example, sleep deprivation has been linked to altered pubertal timing in human and animal data, with the gut microbiome proposed as one mediating pathway (112). In adolescent animal models, sleep deprivation-related gut dysbiosis has also been associated with systemic metabolic changes and impaired reproductive function (113). These findings do not directly establish a pathway to adolescent depression, but they reinforce the broader point that sleep and circadian disruption can interact with gut microbial, endocrine, and metabolic systems during sensitive developmental windows.

Overall, current evidence supports sleep and circadian health as potentially modifiable factors relevant to gut microbiota homeostasis, neuroimmune balance, and long-term mental health during adolescence (108, 109, 114).

## 6 Clinical interventions and future directions

Having systematically reviewed the gut microbiota–inflammation–brain crosstalk mechanisms implicated in adolescent depression, this chapter turns to their translational implications. The preceding sections highlighted several potentially modifiable pathological processes, including immune dysregulation, hypothalamic–pituitary–adrenal (HPA) axis hyperactivity, impaired intestinal barrier function, and disturbances in tryptophan, short-chain fatty acid (SCFA), and neurotransmitter-related metabolism. These mechanistic insights have stimulated increasing academic and clinical interest in non-pharmacological, mechanism-informed interventions targeting the microbiota–gut–brain axis (MGBA) (115, 116). However, given the developmental complexity of adolescence and the heterogeneity of depression, such approaches should currently be viewed as promising adjunctive or preventive strategies rather than established standalone treatments.

This chapter focuses on two intervention modalities that have received particular attention: psychobiotics and dietary interventions. It critically evaluates the current evidence, highlights the limits of extrapolating adult findings to adolescents, and outlines future directions for precision, developmentally sensitive MGBA-based interventions.

### 6.1 New intervention strategies targeting the gut–brain axis: from probiotics to dietary patterns

Psychobiotics are commonly defined as live microorganisms that, when administered in adequate amounts, may confer mental health benefits through interactions with the host microbiota–gut–brain axis (117). In recent years, randomized controlled trials (RCTs) and meta-analyses have examined whether probiotic or prebiotic supplementation can reduce symptoms of depression and anxiety. Overall, available evidence suggests potential benefits, particularly for depressive symptoms, but the magnitude and consistency of effects vary substantially across studies (117, 118). This heterogeneity indicates that psychobiotic effects are unlikely to be uniform across populations and may depend on specific strains, combinations of strains, dosage, treatment duration, baseline microbial ecology, diet, medication exposure, and clinical phenotype.

Mechanistically, psychobiotics may influence depressive symptoms through several pathways that overlap with those discussed in previous chapters. First, beneficial bacteria and their metabolites, especially SCFAs, can modulate systemic and intestinal inflammation, strengthen epithelial barrier integrity, and reduce the translocation of pro-inflammatory microbial products such as lipopolysaccharide. Second, probiotic-related shifts in microbial metabolism may influence tryptophan availability and the balance between serotonin-related and kynurenine-pathway metabolites. Third, the gut microbiota can signal to the central nervous system through vagal afferents, enteroendocrine signaling, immune mediators, and HPA-axis modulation. These mechanisms provide a plausible biological rationale for psychobiotic interventions, although direct causal evidence in adolescents with diagnosed major depressive disorder remains limited.

Evidence in younger populations is encouraging but still preliminary. A systematic review and meta-analysis focusing on young people reported that psychobiotic interventions may reduce anxiety symptoms, suggesting potential relevance for youth mental health (119). Other systematic reviews have indicated that psychobiotic interventions may influence cognitive functioning and emotional behavior in children and adolescents (120), and that probiotic supplementation can be associated with changes in gut microbiota composition in pediatric neuropsychiatric conditions (121). Nevertheless, most available youth studies are heterogeneous in age range, target symptoms, probiotic formulations, and outcome measures, and many are not specifically designed for adolescents with clinically diagnosed depression. Therefore, current findings should be interpreted as evidence of potential rather than definitive clinical efficacy.

Specific clinical trials further illustrate both the promise and the limitations of this field. For example, an RCT in university students with test anxiety found that supplementation with Lactobacillus plantarum JYLP-326 was associated with improvements in anxiety, depressive symptoms, and insomnia, alongside changes in gut microbiota and metabolism (122). Although relevant to youth and young adults, this population differs from early- and mid-adolescent patients with major depressive disorder. In adult patients with depression, probiotics used as adjunctive treatment have shown acceptable tolerability and possible therapeutic effects (123). Earlier double-blind RCT evidence also suggested that supplementation with Lactobacillus acidophilus and Bifidobacterium bifidum improved Beck Depression Inventory scores and metabolic markers in adults with major depressive disorder (124). However, not all probiotic-containing regimens have demonstrated clear superiority over placebo (125), reinforcing the need to identify responder subgroups and optimize intervention protocols.

A key biological challenge is that the host’s baseline gut microbiota represents a complex and dynamically balanced ecosystem. Exogenous probiotics do not enter an empty niche; rather, they must interact with established microbial communities, diet-derived substrates, host immunity, intestinal physiology, and medication exposures. Their effects may therefore be transient, context-dependent, or strain-specific. This ecological perspective may help explain why probiotic effects are more robust in some individuals than in others and why future trials should incorporate baseline microbiome profiling, dietary assessment, inflammatory biomarkers, and metabolomic readouts.

Compared with supplementation using selected strains, dietary intervention represents a broader and more sustained ecological approach to modulating the gut microbiota, inflammatory tone, and metabolic signaling (16, 126, 127). Observational studies consistently suggest that healthier dietary patterns are associated with a lower risk of depressive outcomes (128), whereas poor-quality diets, including high consumption of junk food or ultra-processed foods, are associated with greater psychological distress in children and adolescents (129). Studies of food groups and mental disorders in general populations also support links between diet quality and depression or anxiety risk (130). However, observational evidence cannot determine causality and is vulnerable to confounding by socioeconomic status, physical activity, sleep, family environment, and pre-existing mental health status.

Among dietary patterns, the Mediterranean diet has received particular attention because it is rich in fruits, vegetables, legumes, whole grains, nuts, fish, olive oil, fiber, and polyphenols. These components may promote SCFA-producing bacteria, improve gut barrier integrity, and reduce systemic inflammation. A review focusing on adolescent depression identified Mediterranean-style dietary patterns as a promising strategy for prevention and adjunctive management, partly because of their anti-inflammatory and prebiotic properties (102). However, direct RCT evidence in adolescents remains limited, and most clinical trials have been conducted in adults.

Adult trials nevertheless provide important proof-of-concept evidence. The HELFIMED trial showed that a Mediterranean-style dietary intervention supplemented with fish oil improved diet quality and mental health outcomes in adults with depression (131). Meta-analytic evidence also supports the potential of anti-inflammatory dietary patterns for improving depressive symptoms (132). In addition to whole dietary patterns, supplementation with specific nutrients or combined regimens has been explored. For example, probiotic and vitamin D co-supplementation improved clinical symptoms and mental health-related outcomes in an adult clinical population with migraine (133), although this finding cannot be directly generalized to adolescent depression. Overall, current evidence supports diet as a biologically plausible and clinically relevant target, but adolescent-specific intervention studies are still scarce (134).

Therefore, dietary recommendations for adolescents with depressive symptoms should be framed carefully. Rather than presenting diet as a replacement for evidence-based psychiatric care, it may be more appropriate to consider dietary optimization as part of a multimodal care plan that includes psychotherapy, family and school support, physical activity, sleep regularity, and, when indicated, pharmacotherapy. Developmentally appropriate dietary interventions should also account for autonomy, family food environments, cultural dietary patterns, body image concerns, eating disorder risk, and socioeconomic barriers to accessing healthy foods.

### 6.2 Challenges, limitations, and future perspectives

Despite the growing enthusiasm surrounding MGBA-targeted interventions, several limitations must be acknowledged. First, many human studies linking diet, microbiota, inflammation, and depression are cross-sectional or observational. This makes causal inference difficult and leaves unresolved the “chicken-or-the-egg” question: does an altered microbiota and unhealthy dietary pattern contribute to depression, or does depression, through appetite changes, sleep disruption, reduced activity, stress physiology, and medication use, reshape the microbiota and dietary behavior? Recent large-scale observational and microbiome studies are beginning to address these relationships, but causal pathways remain incompletely defined (135, 136).

Second, clinical trials and meta-analyses often show substantial heterogeneity. This may arise from differences in probiotic strains, dietary protocols, intervention duration, adherence, age, sex, pubertal stage, baseline symptom severity, medication exposure, and outcome measures (137, 138). Adolescence adds additional complexity because sex hormones, pubertal maturation, school stress, sleep timing, peer relationships, and family environments may all interact with the gut microbiota and inflammatory regulation. As a result, interventions that are effective in adults may not have the same efficacy, acceptability, or biological effects in adolescents.

Third, descriptive microbiota studies in psychiatric disorders have not yielded fully consistent microbial signatures. Systematic reviews of major depressive disorder, bipolar disorder, schizophrenia, anxiety, and depression indicate that although reduced microbial diversity, altered SCFA-producing taxa, and changes in inflammatory or metabolic pathways are often reported, specific taxa-level findings vary across cohorts (139, 140). These inconsistencies may reflect methodological differences in sequencing platforms, bioinformatic pipelines, sample handling, geography, diet, medication use, and clinical heterogeneity. Consequently, microbiota-based interventions should not yet be based on a presumed universal “depression microbiome.”.

Future research should move toward more precise, developmentally informed, and mechanism-based approaches. One priority is to conduct well-powered longitudinal studies and RCTs specifically in adolescents with depressive symptoms or clinically diagnosed depression. These studies should integrate microbiome sequencing with metabolomics, inflammatory markers, HPA-axis measures, dietary assessment, sleep and activity monitoring, and standardized psychiatric outcomes. Such multi-layered designs would help determine whether changes in microbial composition or microbial metabolites mediate clinical improvement, rather than merely correlating with it.

A second priority is patient stratification. Adolescents with depression are unlikely to represent a biologically homogeneous group. Microbiota profiles, inflammatory status, tryptophan metabolism, sex, pubertal stage, dietary intake, antibiotic exposure, and early-life stress may all shape treatment response. Biomarker-guided approaches could therefore help identify which adolescents are most likely to benefit from a specific probiotic, prebiotic, synbiotic, dietary pattern, or combined intervention. Studies linking adolescent depression with gut microbiota and tryptophan-derived metabolites provide an important foundation for this direction (141).

A third direction involves the development of more targeted interventions. Future MGBA-based therapies may include next-generation probiotics, defined microbial consortia, postbiotics, prebiotic fibers, fermented foods, polyphenol-rich dietary strategies, and natural product-based approaches targeting specific microbial or inflammatory pathways (23). However, these interventions will require careful safety evaluation in adolescents, particularly because the adolescent brain, endocrine system, immune system, and microbiota are still developing.

Finally, future clinical practice will likely emphasize participatory and multimodal care. Adolescents should not be passive recipients of microbiome-directed interventions; rather, they should be supported in actively managing diet, sleep, physical activity, stress, and treatment adherence in collaboration with families, schools, and healthcare providers. In this sense, MGBA-targeted strategies align with the broader model of predictive, preventive, personalized, and participatory medicine. The ultimate goal is not to replace established psychiatric treatments, but to expand the therapeutic toolkit by adding biologically informed, acceptable, and scalable strategies for prevention, early intervention, and adjunctive care.

In summary, psychobiotics and dietary interventions represent promising but still developing approaches for modulating the microbiota–inflammation–brain network in adolescent depression. Current evidence supports biological plausibility and potential clinical benefit, especially as adjunctive strategies, but adolescent-specific causal and interventional evidence remains insufficient. Future studies that combine rigorous clinical trial design with multi-omics profiling and developmental stratification will be essential for translating gut–brain axis science into safe, precise, and effective interventions for young people.

## 7 Conclusion

Adolescent depression is a major and growing public health concern. Although existing treatments, including psychotherapy and pharmacotherapy, remain beneficial for many young people, their overall effectiveness is variable, and important questions remain regarding tolerability, developmental suitability, and long-term outcomes in this age group. These limitations highlight the need to investigate additional biological mechanisms that may help explain vulnerability during adolescence and inform more developmentally appropriate prevention and intervention strategies.

This review has proposed an integrative framework in which adolescence may represent a potential “dual-sensitive period, ” characterized by the partially overlapping maturation of two highly plastic systems: the brain and the gut microbiota. During this developmental stage, neural circuits involved in emotion regulation—particularly prefrontal-limbic networks—undergo substantial remodeling, while the gut microbial ecosystem remains responsive to hormonal, dietary, environmental, and behavioral influences. The temporal overlap of these developmental processes may create a window of increased susceptibility in which perturbations of the microbiota-gut-brain axis (GBA) could interact with ongoing neurodevelopment and stress regulation. At present, however, this framework should be regarded as a biologically plausible and conceptually useful model rather than a definitively established mechanism of adolescent depression.

Across the evidence reviewed, several interrelated pathways appear especially relevant. Current data support the possibility that gut dysbiosis may contribute to low-grade peripheral inflammation, altered intestinal barrier integrity, and abnormal signaling to the brain through immune, neural, endocrine, and metabolic routes. These signals may, in turn, influence microglial function, synaptic remodeling, neurotransmitter-related metabolism, and hypothalamic-pituitary-adrenal (HPA) axis responsivity. In particular, the hypothesis that inflammation-associated microglial dysregulation may contribute to maladaptive synaptic pruning provides one potential mechanistic bridge between gut-derived disturbances and altered maturation of emotion-related neural circuits. Nevertheless, much of the strongest mechanistic evidence for these pathways still derives from animal models or adult populations, and direct causal evidence in human adolescents remains limited.

This review has also considered sex differences through the conceptual lens of the microgenderome. The marked increase in depression prevalence among females after puberty suggests that sex-dependent biological processes may be relevant to adolescent vulnerability. Interactions among sex hormones, the gut microbiota, immune signaling, and intestinal and blood-brain barrier function may represent one component of this pattern. However, these mechanisms should not be viewed in isolation or as a complete explanation for sex differences in depression. Rather, they likely operate alongside broader developmental influences, including genetic factors, epigenetic remodeling, psychosocial stressors, family and peer environments, and sociocultural context.

Environmental exposures common in contemporary adolescence may further modulate this system. Diet quality, antibiotic exposure, and sleep or circadian disruption each have the potential to influence gut microbial ecology, inflammatory tone, metabolic signaling, and stress responsivity. These factors may therefore act as important perturbing influences on the adolescent GBA and may amplify vulnerability in susceptible individuals. However, the available evidence does not support a simple one-directional causal interpretation, and future work will need to clarify timing, dose, reversibility, and individual susceptibility.

From a translational perspective, the GBA is of particular interest because it may offer modifiable targets for prevention and adjunctive care. Psychobiotics, dietary strategies, and other microbiota-informed interventions have shown promising early signals in preclinical research and selected human studies. However, based on current evidence, these approaches should be viewed as developing adjunctive or preventive strategies rather than established standalone treatments for adolescent depression. Their clinical utility will depend on stronger adolescent-specific evidence, better characterization of responder subgroups, and more rigorous integration of psychiatric, microbiological, immunological, metabolic, and developmental measures.

Several priorities emerge for future research. First, there is a need for large, well-characterized longitudinal studies that follow young people across the pubertal transition and integrate microbiome profiling with metabolomics, inflammatory markers, neuroendocrine measures, neuroimaging, sleep and dietary assessment, and standardized psychiatric phenotyping. Such designs will be essential for distinguishing correlation from causation and for determining whether microbiota-related signals mediate depressive trajectories or treatment response. Second, future intervention studies should focus more specifically on adolescents rather than extrapolating from adult populations. Third, stratified and biomarker-informed approaches may eventually help identify subgroups of adolescents most likely to benefit from particular microbiota-targeted or dietary interventions, but such applications are not yet ready for routine clinical implementation.

At the level of clinical care and public health, the current state of evidence supports a balanced and pragmatic conclusion. Healthy diet, regular sleep, appropriate antibiotic stewardship, physical activity, and supportive psychosocial environments are already important components of adolescent health and may also be relevant to maintaining microbiota-gut-brain homeostasis. While these factors should not be overstated as microbiome-specific treatments for depression, they represent practical and potentially synergistic elements of broader preventive and multimodal care.

In summary, this review argues that the gut-brain axis deserves serious consideration as an important candidate interface linking environment, development, immunity, metabolism, and mental health during adolescence. Current evidence supports biological plausibility for a role of the microbiota-inflammation-brain network in adolescent depression, but key mechanistic, causal, and clinical questions remain unresolved. A more precise understanding of these relationships may ultimately broaden how adolescent depression is conceptualized and managed, while helping to advance safer, developmentally informed, and more personalized strategies for prevention, early intervention, and adjunctive treatment.
